# Gustatory Cortex Is Involved in Evidence Accumulation during Food Choice

**DOI:** 10.1523/ENEURO.0006-22.2022

**Published:** 2022-05-17

**Authors:** Ali Ataei, Arash Amini, Ali Ghazizadeh

**Affiliations:** Electrical Engineering Department, Sharif University of Technology, Tehran 1458889694, Iran

**Keywords:** EEG energy, EEG-informed fMRI analysis, food choice, gustatory cortex, value-based decision-making

## Abstract

Food choice is one of the most fundamental and most frequent value-based decisions for all animals including humans. However, the neural circuitry involved in food-based decisions is only recently being addressed. Given the relatively fast dynamics of decision formation, electroencephalography (EEG)-informed fMRI analysis is highly beneficial for localizing this circuitry in humans. Here, by using the EEG correlates of evidence accumulation in a simultaneously recorded EEG-fMRI dataset, we found a significant role for the right temporal-parietal operculum (PO) and medial insula including gustatory cortex (GC) in binary choice between food items. These activations were uncovered by using the “EEG energy” (power 2 of EEG) as the BOLD regressor and were missed if conventional analysis with the EEG signal itself were to be used, in agreement with theoretical predictions for EEG and BOLD relations. No significant positive correlations were found with higher powers of EEG (powers 3 or 4) pointing to specificity and sufficiency of EEG energy as the main correlate of the BOLD response. This finding extends the role of cortical areas traditionally involved in palatability processing to value-based decision-making and offers the “EEG energy” as a key regressor of BOLD response in simultaneous EEG-fMRI designs.

## Significance Statement

Choosing what to buy at a local grocery or to eat from a diner’s menu involves decision-making based on internal states and past memories. It is shown such food choices can be behaviorally governed by the same evidence accumulation processes underlying other forms of decision-making. A previous investigation using simultaneous electroencephalography (EEG)-fMRI implicated motor related areas in posterior medial frontal cortex (pMFC) in the process. Here, by using an improved methodology, we uncover significant evidence accumulation activity within the Gustatory cortex (GC) itself. Given the involvement of GC in gustatory imagery in addition to primary taste processing, these results suggest the intriguing possibility that during food choice the low-level sensory taste information may be conjured up to guide deliberation between appetitive options.

## Introduction

The choice of what to eat is probably one of the most common and yet most basic forms of decision-making in the Animalia kingdom. This decision-making problem like any other, requires deliberation to commit intentions in favor of one choice to the exclusion of others. In decision-making with uncertain sensory information (also known as perceptual decision-making), this process is often modelled with a drift-diffusion process supposedly representing evidence accumulation in favor of a given option (Britten et al., 1996; Mazurek et al., 2003; Gold and Shadlen, 2007; Hanks et al., 2015). Neural correlates of perceptual decision-making is found in a couple of brain areas most notably the lateral intraparietal sulcus (LIP) and the prefrontal cortex (Gold and Shadlen, 2007; Pisauro et al., 2017). The accumulation of evidence during perceptual decision-making is also observed in human electroencephalography (EEG; Philiastides et al., 2014). In value-based decision-making however, the evidence accumulation is done not on the momentary external evidence but on the mnemonic internal variables representing the subjective value of choice items (Bakkour et al., 2019). More specifically, if we focus on the appetitive values of food items, we shall note that this type of subjective value depends on both the internal states of the subjects and their previous experience. Representations of value memory are reported in a couple of temporal and prefrontal cortices as well as in some basal regions in monkeys and humans (Anderson et al., 2014; Kim et al., 2015; Ghazizadeh et al., 2020; Ghazizadeh and Hikosaka, 2021).

Recent studies have extended the drift diffusion model of choice to value-based decision by relating an item’s subjective value to the drift term in the decision variable (DV; Krajbich et al., 2010; Milosavljevic et al., 2010). Given the relatively rapid evolution of DV in time, neural correlates of such a process have to be searched for by methods with sufficient temporal resolution such as single unit electrophysiology or EEG. In particular, EEG studies have found neural correlates of value-based decision-making across centro-parietal electrodes reflected in the raw EEG or γ band signals (Polanía et al., 2014; Pisauro et al., 2017). However, the low spatial resolution of EEG prevents accurate localization of brain loci for value-based evidence accumulation. One work-around is to use simultaneous EEG-fMRI which combines the localization strength of fMRI with high temporal resolution of EEG (Pisauro et al., 2017). Indeed, with this technique, Pisauro et al. (2017) found EEG signal correlates of DV in a value-based decision-making task and then used these EEG correlates as a regressor on the BOLD responses (EEG-informed fMRI analysis) to find brain regions involved. Using this method, signatures of evidence accumulation was found in the posterior-medial frontal cortex (pMFC). But because of the highly nonlinear mapping from electric potentials to the BOLD signal, the raw EEG may not be the best regressor for BOLD in an EEG-informed fMRI analysis.

Here, we argue that from a theoretical standpoint based on physics of EEG and fMRI, a quadratic relation between BOLD and EEG (i.e., EEG energy) may be more accurate as a first-order approximation. This conjecture is in agreement with previous reports that suggest a linear relation between the mean power of event-related EEG sources and the neural efficacy (input of vascular system for BOLD response; Wan et al., 2006) or between EEG energy across various frequency bands and the BOLD signal (de Munck et al., 2009; Scheeringa et al., 2009; Sato et al., 2010). Notably, reanalysis of simultaneous EEG-fMRI data recorded during a value-based decision-making task with EEG energy as the BOLD regressor, revealed significant activations in cortical areas involved in palatability processing including the insula and operculum which were missed in previous analysis with raw EEG signal.

## Materials and Methods

### Data and analysis

We used the open-access simultaneous EEG-fMRI data associated with Pisauro et al. (2017), https://openneuro.org/datasets/ds001219/versions/1.0.0. The data were partly preprocessed which included motion-correction, slice-time correction, high-pass filtering (>100 s) and spatial smoothing (8-mm FWHM). We performed the remaining proceeding preprocessing steps similar to (Pisauro et al., 2017) except those described below.

### fMRI preprocessing

We used FSL to register the cleaned EPI images to the MNI space just as previously described (Pisauro et al., 2017) using six-parameter rigid body transformation and the nonlinear registration tool, except for subject number 20, for whom we used 12 parameter affine transformation to map his EPI to his structural image, because of a need for scaling in this case. Finally, the BOLD signal in each voxel is transformed to percentage of change with respect to time average of that voxel for the subsequent analyses.

### Building EEG and EEG energy regressors

We used the raw signal of the best EEG electrode in the decision time period as described previously (Pisauro et al., 2017, which takes zero value outside the decision periods; Extended Data Fig. 3-1*b*). After convolving the EEG regressor with the HRF, we subsampled this signal in intervals equal to the fMRI repetition time (here, TR = 2.5 s) and replaced the signal at each TR by its temporal mean within that TR. The EEG signal previously described (Pisauro et al., 2017) was first subsampled to 50-ms resolution, then convolved with HRF and then subsampled to fMRI TR. Our downsampling method does not result in significant difference from that by Pisauro et al. (2017), since the signal was smoothed because of convolution with the HRF. We did the same procedure but with the square of the EEG signal to build the “EEG energy” regressor. We demeaned all regressors. For the normalized regressors, we also divided them by their SD.

**Figure 1.**
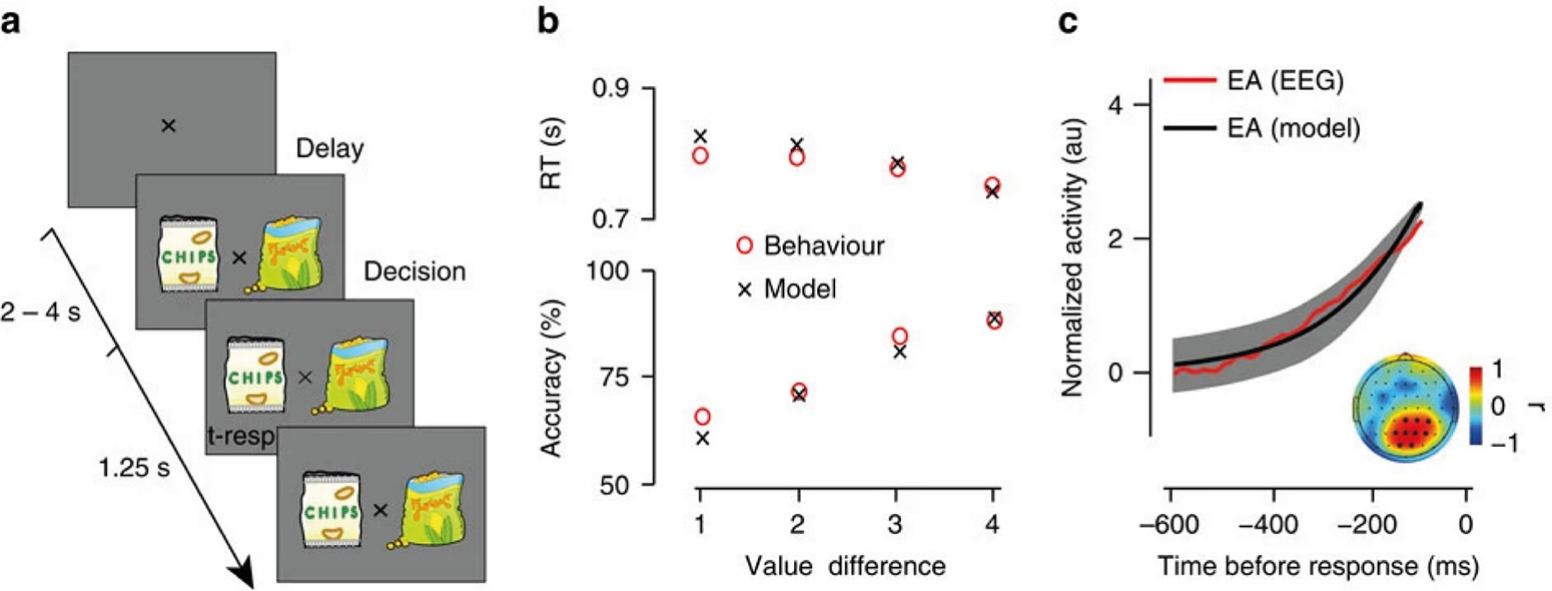
Task design, behavioral, and modeling results and EEG. ***a***, Schematic representation of the experimental paradigm. After a variable delay (2–4 s), two stimuli (snack items) were presented on the screen for 1.25 s, and participants had to indicate their preferred item by pressing a button. The central fixation dimmed briefly when a response was registered. Snack stimuli shown here are for illustration purposes only. Participants viewed real branded items during the experiments. ***b***, Behavioral performance (red circles) and modeling results (black crosses). Participants’ average (*N* = 21) reaction time (RT) and accuracy (top and bottom, respectively) improved as the value difference (VD) between the alternatives increased. An SSM that assumes a noisy moment-by-moment accumulation of the Vd signal fit the behavioral data well. ***c***, Average (*N* = 21) model predicted evidence accumulation (EA; black) and EEG activity (red) in the time window leading up to the response (on average, 600–100 ms before the response), arising from a centroparietal electrode cluster (darker circles in the inset) that exhibited significant correlation between the two signals. Shaded error bars represent standard error across participants (reproduced with permission from Pisauro et al., 2017).

10.1523/ENEURO.0006-22.2022.f3-1 Extended Data Figure 3-1 The regressors used in fMRI analyses. a, The four nuisance regressors; the visual onset, the value difference, the reaction time, and the visual offset regressors. b, The regressors of interest; the raw EEG and the EEG energy regressors. Download Figure 3-1, TIF file.

### fMRI analysis and the generalized linear models (GLMs)

We did the GLM analyses in AFNI 20.2.05. We used step-wise GLM analyses to gauge robustness of our findings and to overcome the multicollinearity between various EEG-driven regressors (various powers of EEG) as well as the correlation between the regressor of interest and the nuisance regressors. For most stringent analyses, we used EEG raw signal in the primary GLM and the higher powers in the subsequent GLMs to ensure explaining brain activation over and beyond raw EEG. GLM1 is just same as the main GLM analysis by Pisauro et al. (2017) and consists of the three nuisance regressors and the raw EEG regressor. GLM 2 is a regression of the residuals of GLM1 over the “EEG energy.” This paradigm is in favor of the null hypothesis (sufficiency of raw EEG) and lets it explain as much variance in the fMRI data and only leaves the orthogonal components to be explained by the new regressor, i.e., EEG energy. Since visual stimulus offset timing is close to the reaction times for food choice decision (<1 s), it can be a serious confound for the decision correlates observed in brain activation. To control for this effect, GLM3 is performed. This GLM is similar to GLM1, but with an added nuisance regressor accounting for the stimulus offset. Once again GLM4 regresses the residuals of GLM3 over the EEG energy to ensure robustness of findings with respect to inclusion of vstim-off. Simultaneous regression using both raw EEG and EEG energy is also performed (GLM6) over residuals of GLM5 which only consists of the nuisance regressors. We have also performed similar analyses replacing vstim-off with a boxcar function for the duration of visual stimulus in GLMs 7–9. Since the total EEG power substantially differs from subject to subject, we repeated similar analyses in GLMs 10–11, using normalized EEG-driven regressors (normalizing EEG regressors in each subject by their SD). Higher order powers of EEG were also investigated. GLM12 regresses the residuals of GLM3 over the third power of EEG signal. In order to test the fourth power of EEG signal (GLM13), we used residuals of GLM4 rather than GLM3, because of the high correlation between powers 2 and 4. Details of all regressions done in this study can be found in Table 1.

**Table 1:** Summary of all GLMs used in the study

GLM index	Signal to regress	Regressors
GLM1	BOLD	vstim – VD – rt – EEG
GLM2	Residual of GLM1	EEG energy
GLM3	BOLD	vstim – VD – rt – vstim off – EEG
GLM4	Residual of GLM3	EEG energy
GLM5	BOLD	vstim – VD – rt – vstim off
GLM6	Residual of GLM5	EEG, EEG energy
GLM7	BOLD	vstim (onset) – VD – rt – vstim boxcar
GLM8	Residual of GLM7	EEG
GLM9	Residual of GLM7	EEG energy
GLM10	Residual of GLM5	EEG (normalized)
GLM11	Residual of GLM5	EEG energy (normalized)
GLM12	Residual of GLM3	EEG pow3
GLM13	Residual of GLM4	EEG pow 4

The model for each primary GLM is as:

(1) Y=βX + r.

Where, *Y* is the time series of the normalized BOLD response of a single voxel for *T* time samples, *X* is a n×T design matrix with rows representing *n* regressors. β is a 1×n vector, containing the regression weights for each regressor for this particular voxel and r is the 1×T residual of this regression.

The model for each secondary GLM (second step of step-wise GLM) is as:

(2) $$r=\beta_{2}X_{2}+\varepsilon .$$

Where, r is the residual from the primary GLM for a specific voxel, $X_{2}$ is the design matrix for the regressors of interest and ϵ is the regression residual.

We performed all our GLMs in AFNI via “3dREMLfit.” For group-level analysis, we used “3dttest++.” The group-level activation maps were then masked by the gray matter mask associated with the standard MNI brain with resolution of 2 mm (results for the raw EEG regressor were not masked to make them comparable with Pisauro et al., 2017). By applying 3dFWHMx on these group-level residuals, we estimated the parameters for the non-Gaussian spatial autocorrelation function of the fMRI noise. Then, using 3dClustSim, we calculated the cluster thresholds for various *p*-values such that the probability of a false positive cluster among the *p*-thresholded clusters is less than α = 0.05. The inflated surfaces are presented using SUMA 20.2.05.

### The lead-field matrix for analysis of correlation of voxels

In this part, we collected whole-brain MRI T1 image for a single subject, using a 3T Siemens scanner with resolution of 1 mm. In order to make a precise estimation of the lead-field, we also captured the EEG electrode coordinates registered to the subjects T1-image using Localite TMS navigator. Both measurements were performed in the National Brain Mapping Laboratory (NBML, Tehran, Iran). Then we calculated the lead-field matrix using “brainstorm” toolbox. We used a custom three-layer BEM head-model and tessellated the cortex to 15,000 vertices and assumed dipoles perpendicular to the cortex surface.

### Code accessibility

The lead-filed matrix and the simulation code as well as the fMRI analysis codes are available at https://github.com/poyaata/code-data.

## Results

We re-examined the simultaneous EEG-fMRI data previously published (Pisauro et al. (2017)), in which subjects were asked to choose between pairs of previously rated snack items and to indicate their choice with a button press (Fig. 1*a*). Each trial began with display of a fixation point for a random time in the range 2–4 s, and the subjects were asked to maintain the fixation point. Then, two randomly selected food items were displayed to the right and to the left of the fixation point for 1.25 s, and the subject had to respond during this period. The difficulty of the decision was controlled by the value difference (VD) in the ratings of the presented items. Before the experiment and outside the scanner, each subject was asked to rate 80 snack items with real scores in the range of (−5,5) based on his/her subjective value. The EEG electrode that best matched “theoretical prediction of a dynamical sequential sampling model (SSM) fitted to the behavioral data of each subject” was used as a regressor against the BOLD signal in all voxels for localization of brain regions supporting decision-making in this task (Fig. 1*b*,*c*; for further details on the task and previous findings, see Pisauro et al., 2017).

Initial analysis of this data by Pisauro et al. (2017) using the electrode whose raw EEG signal correlated best with the evidence accumulation model prediction, revealed a significantly positive cluster in pMFC. In the original generalized linear regression model (GLM), raw EEG was used as the signal of interest along with three nuisance factors including visual stimulus onset, the value difference (of food items) and the (subject’s) reaction time (Extended Data Fig. 3-1).

As suggested previously (Wan et al., 2006) and based on physiological relationship between BOLD and energy consumption in a given region, we hypothesized that the instantaneous energy of a desired EEG electrode should be a more natural regressor against BOLD response.

### Modelling the relation between EEG and BOLD

EEG and other extracellular measurements are the electric potentials associated with volume conduction of current dipoles arising from a bulk of activated neurons (Buzsáki et al., 2012). These dipoles arise from ion displacements across the cell membrane. The dissipated energy through these displacements would be proportional to the square of the membrane voltage, $V^{2}$. Moreover, the electrical work performed by the active pumps can also be shown to be proportional to $V^{2}$, since a linear relation between the pump’s current and the membrane voltage in a wide range of pump’s activity is previously reported (Nakao and Gadsby, 1989). Therefore, the energy used in a voxel may be considered proportional to the square of “the electric potential or the magnitude of the current dipole” associated with this voxel. In particular, the electric potential for EEG electrode *i*, $e_{i}(t)$ can be written as the weighted sum of the dipole magnitudes from voxels across the brain:

(3) $$e_{i}(t)=\sum_{j=1}^{L}W_{ij}v_{j}(t)+n_{i}(t)\;.$$

Where, L is the number of voxels (dipoles), $v_{j}(t)$ is the dipole magnitude associated with voxel *j*, $W_{ij}$ are the lead-field weights from voxels to the EEG electrodes and $n_{i}(t)$ is the noise present in EEG electrode *i*. The energy of this signal over a time interval of *T* equals:

(4) $$\varepsilon_{i}=\int_{T}e_{i}^{2}(t)=\sum_{j=1:L}W_{ij}^{2}\int_{T}v_{j}^{2}(t)+\sum_{j,k;j\neq k}W_{ij}W_{ik}\int_{T}v_{j}(t).v_{k}(t)+\varepsilon_{noise}.$$

While the first summation in the right-hand side (R.H.S.) of Equation 4 is a weighted sum of the energies consumed in voxels, the second term is a summation of the voxels’ correlations. While the first term is strictly positive, the second term can be suppressed because of positive and negative correlations across voxels. The noise term $n_{i}(t)$ is assumed to be orthogonal to the dipole time series. Thus, as a first order approximation, EEG energy can be considered as a weighted sum of dipole energies in voxels which is the first term in Equation 4. The error in this approximation can be quantified as the ratio of the second summation in Equation 4 with respect to the whole sum as parameter z:

(5) $$z=\frac{\left|\sum_{j,k;j\neq k}W_{ij}W_{ik}\int_{T}v_{j}(t).v_{k}(t)\right|}{\sum_{j=1:L}W_{ij}^{2}\int_{T}v_{j}^{2}(t)+\sum_{j,k;j\neq k}W_{ij}W_{ik}\int_{T}v_{j}(t).v_{k}(t)}.$$

In order to quantify the amount of error arising from ignoring the second term in Equation 4, we conducted multiple simulations for various correlation magnitudes between the sources using a real lead-field matrix (for details of lead-field construction, see Materials and Methods). Specifically, we considered 20 active sources randomly distributed in the brain and produced the associated coherence matrix between these sources using a truncated Gaussian distribution. The overall correlation level between sources was controlled with the variance of this Gaussian distribution. We considered four levels of overall coherences and performed 10,000 simulations for each level.

**Figure 2.**
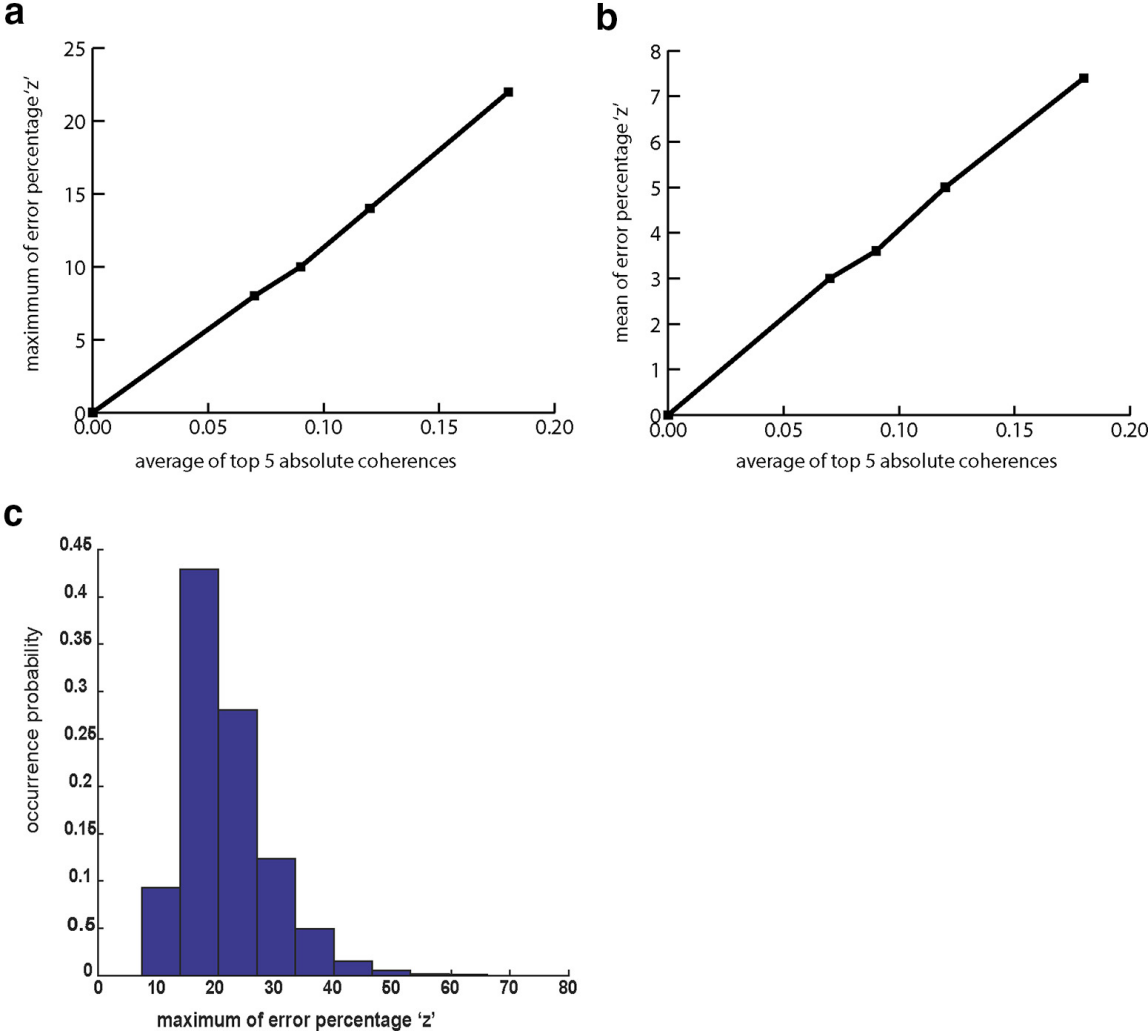
EEG energy of electrodes is a good correlate of energy consumption across voxels for modest levels of dipole coherences. ***a***, Effect of dipole coherences on the maximum of the error percentage (‘z’) in approximating EEG energy as a sum of voxel BOLD values among 63 EEG channels averaged across simulations. ***b***, Same as ***a*** but for the average of ‘z’ rather than its maximum across EEG electrodes. ***c***, The histogram of the maximum of ‘z’ among EEG channels for the highest coherence level simulated (coherence = 0.2).

In each simulation, we recorded the average of the five highest correlation magnitudes between the sources (as the overall coherence level) as well as the maximum and average of z among the 63 EEG electrodes. Simulations show that while this error term is an increasing function of correlation among dipole sources, nevertheless its maximum among the electrodes is <25% by average (Fig. 2*a*) even for coherences of up to 0.2 among voxels. The coherences below 0.2 are reported previously (Lehmann et al., 2012; Nentwich et al., 2020) and seem relevant even for patients with epilepsy and schizophrenia with high levels of synchrony among regions (Bowyer, 2016).While the maximum of z among EEG electrodes provides the upper-bound of error, the expected value of error tends to be much smaller (<8%) in the same dynamic range of coherences between dipole sources (Fig. 2*b*). Figure 2*c* also indicates that even for the highest correlation level, the maximum of z among electrodes lies most frequently between 10% and 20%.

Therefore, we can approximately write:

(6) $$\varepsilon_{i}[n]=\int_{nT}^{nT+T}e_{i}^{2}(t)=\sum_{j=1:L}M_{ij}\int_{nT}^{nT+T}v_{j}^{2}(t)\;;\;\;M_{ij}=W_{ij}{}^{2}.$$

Obviously the route from the energy consumption in a voxel and the observed BOLD signal has to go through a couple of other steps including neurovascular coupling and blood vessel dynamics which subject this relationship to further nonlinearities and smoothing and can be modelled by detailed biophysical processes such as the Balloon model (Buxton et al., 1998). However, for simplicity, here, we only considered the simple hemodynamic function commonly used in the analysis of BOLD with GLMs. In this case, convolving the two sides of Equation 6 with the hemodynamic function we will have:

(7) $${\tilde{\varepsilon }}_{i}[n]=\varepsilon_{i}[n]*h[n]=\sum_{j=1:L}M_{ij}\left(b_{j}[n]*h[n]\right)\;;\;b_{j}[n]=\int_{nT}^{nT+T}v_{j}^{2}(t).$$

Since $b_{j}\left[n\right]*h[n]$ is assumed to be proportional to the BOLD signal of voxel *j* in the time volume *n*, the R.H.S. of Equation 7 is actually a weighted sum of the BOLD signals.

### EEG energy shows evidence accumulation in cortical regions involved in processing of food palatability

**Figure 3.**
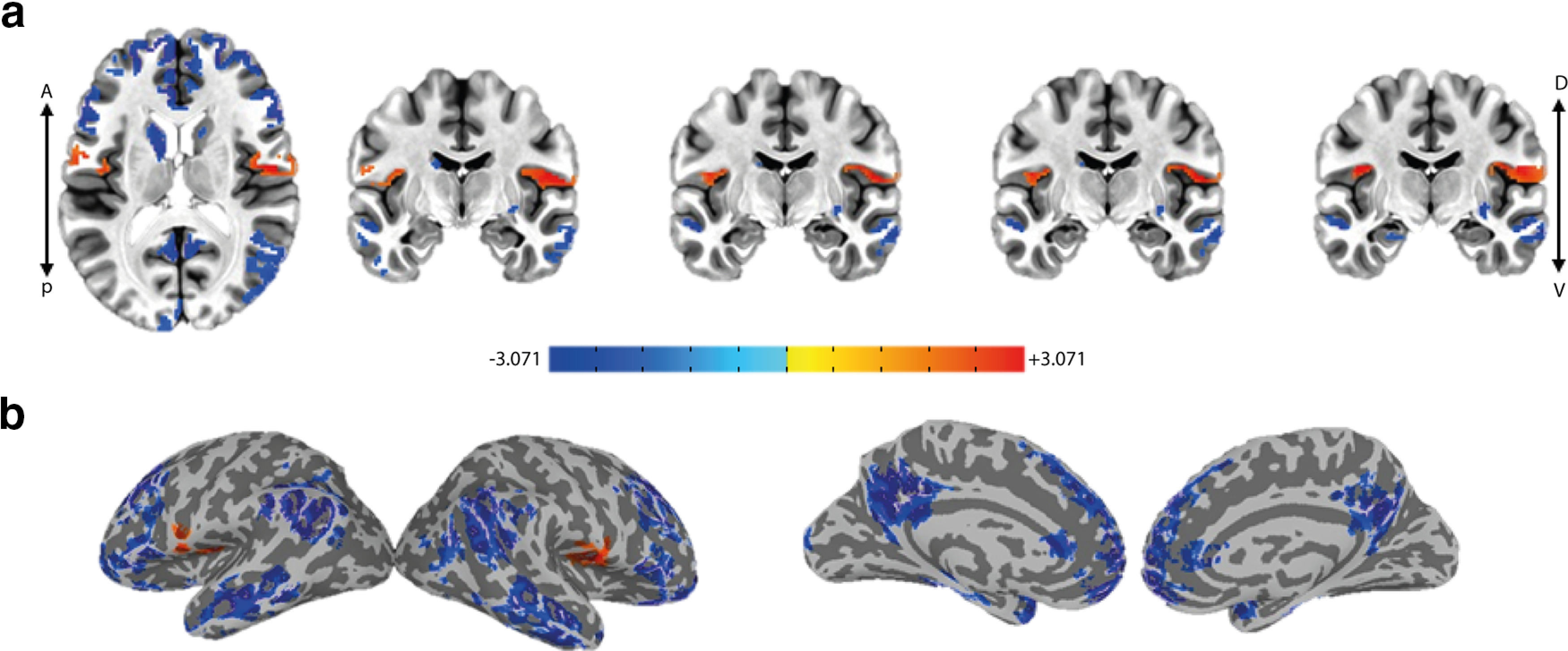
GC shows a significant positive correlation with EEG energy during food choice (GLMs 1–2). Group-average activation map (*t* stats) for the “EEG energy” regressor in GLM2 (step-wise regression on the residuals of original GLM done by Pisauro et al., 2017) showing activity in the bilateral insular, opercular and inferior somatosensory cortices; *p* < 0.05, cluster-corrected (right cluster = 293, left cluster = 218 > threshold = 136); ***a***, axial and multiple coronal views, ***b***, lateral and medial views on the inflated cortex. See Extended Data Figure 3-1 for the illustration of the regressors used in the single-subject GLMs. See Extended Data Figure 3-2 for the activation maps regarding to the nuisance regressors.

To examine whether “EEG energy” explains BOLD fluctuations over and above the three nuisance factors and the raw EEG, we used a step-wise GLM paradigm by first repeating the main GLM analyses previously described (Pisauro et al., 2017; Table 1, GLM1) and then regressing its residuals over the EEG energy regressor (Table 1, GLM2). Interestingly, the activation map for the EEG energy (Fig. 3; *p* < 0.05, cluster-corrected; Table 2) was highly different from the activation map for the raw EEG (Extended Data Fig. 4-1*a*) and showed one significant positive cluster in each hemisphere that included parts of operculum, insula and the inferior somatosensory cortex [collectively referred to as the gustatory cortex (GC) hereafter] as well as several significant negative clusters across frontal, temporal, occipital, and temporoparietal regions.

**Table 2:** Number of voxels and location of peak activity for both positive and negative clusters

Region/cluster	#(voxels)	Hemisphere	Peak X	Peak Y	Peak Z	BA
Energy, GLM2 (+):						
Inferior somatosensory (gustatory cortex)	293	Right	−56	8	14	43/6
Insula (gustatory cortex)	213	Left	4	12	20	13
Energy, GLM4 (+):						
Inferior somatosensory (gustatory cortex)	214	Right	−54	8	14	43/6
Energy, GLM6 (+):						
Inferior somatosensory (gustatory cortex)	213	Right	−54	8	14	43/6
Energy, GLM9 (+):						
Inferior somatosensory (gustatory cortex)	219	Right	−54	8	14	43/6
Energy, GLM11 (+):						
Insula (gustatory cortex)	567	Right	−44	10	20	13
Energy, GLM4 (–):						
Superior frontal gyrus	10945	Right	−18	−56	30	9
Inferior parietal lobe	3351	Right	−48	54	56	40
Supramarginal gyrus	2157	Left	58	54	36	40
Superior temporal gyrus	2048	Left	30	−14	−34	38
Middle temporal gyrus	1668	Right	−52	22	−12	21
Cingulate gyrus	928	Left	12	44	30	31
Cuenus	872	Left	28	90	22	19
Cingulate gyrus	791	Right	−4	44	34	31
Middle occipital gyrus	705	Left	38	64	−2	37
Declive (cerebellum)	683	Right	−34	68	−26	—
putamen	603	Right	−22	−12	0	—
Superior temporal gyrus	528	Right	−44	−10	−20	38
Inferior frontal gyrus	488	Left	28	−10	−18	47
Caudate head	257	Left	8	−4	6	—
Thalamus	150	Right	−14	12	12	—
Culmen (cerebellum)	148	Right	−30	46	−32	—
EEG pow3, GLM12 (–):						
Middle frontal gyrus	275	Left	40	−50	−10	11
Inferior parietal lobe	140	Right	−46	68	48	40
Inferior parietal lobe	135	Left	40	66	48	40

BA, Broadman Area.

10.1523/ENEURO.0006-22.2022.f3-2 Extended Data Figure 3-2 Activation maps for the nuisance regressors (GLM 3). Group-average activation map (t stats) for the nuisance regressors all with p < 0.01 and cluster-corrected: (a) visual onset (vstim) regressor, (b) visual offset (vstim-off) regressor, (c) value difference (Vd) regressor, and (d) reaction time (rt) regressor. Download Figure 3-2, TIF file.

**Figure 4.**
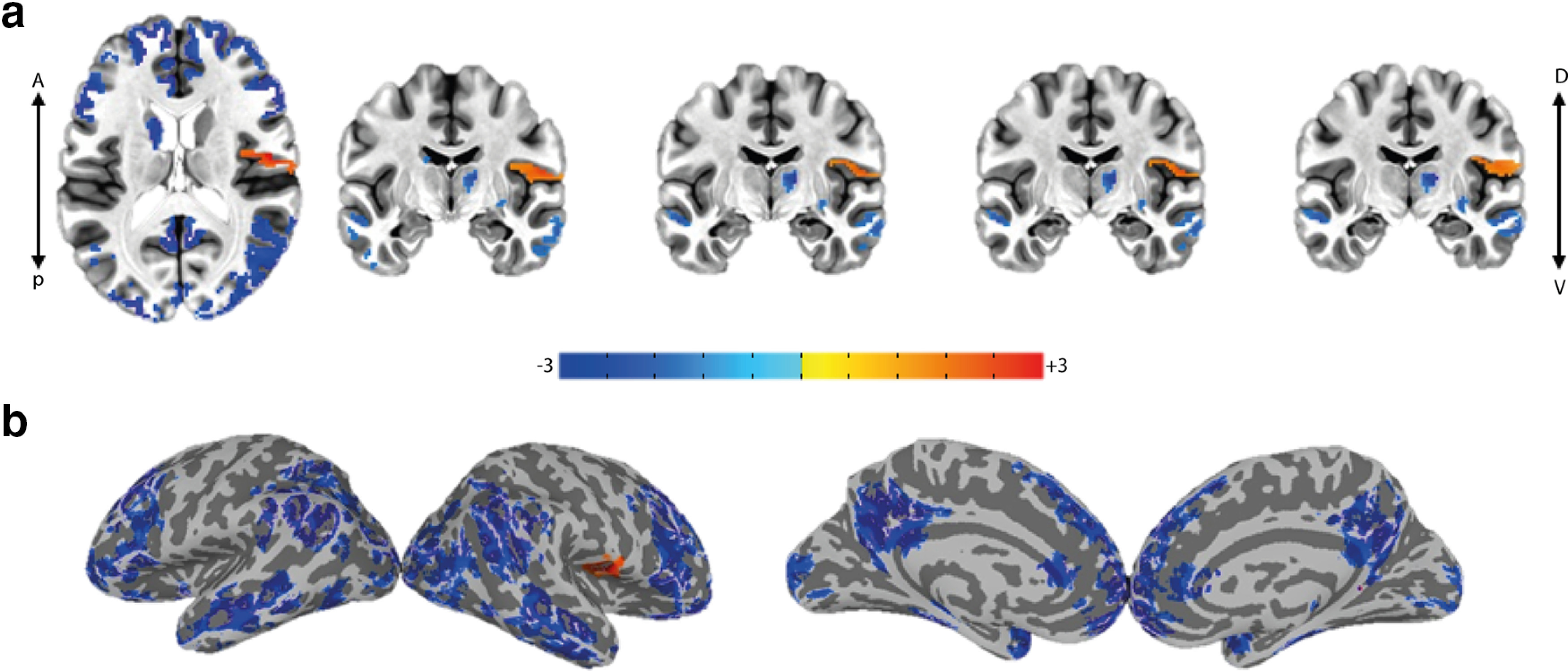
Right GC activation is robust to the addition of visual stimulus offset as a nuisance regressor (GLMs 3–9). Group-average activation map (*t* stats) for the “EEG energy” regressor in GLM4 showing activity in the right insular, opercular and inferior somatosensory cortices (*p* < 0.05, cluster-corrected (cluster = 214 > threshold = 121); ***a***, axial and multiple coronal views, ***b***, lateral and medial views on the inflated cortex. See Extended Data Figure 4-1 to compare the effect of addition of “vstim-off” regressor on the activation map for the raw EEG regressor. See Extended Data Figure 4-2 for a similar result for EEG energy when the raw EEG and EEG energy regressors are simultaneously used in the second-step GLM. See Extended Data Figure 4-3, for activations revealed by the raw EEG and EEG energy if instead of vstim-off, one uses a boxcar function for the duration of visual stimulus.

10.1523/ENEURO.0006-22.2022.f4-1 Extended Data Figure 4-1 Comparing the activity map for the raw EEG regressor with and without considering stimulus offset as a nuisance regressor (GLMs 1, 3) Group-average activation map for the raw EEG regressor (a) without considering “vstim-off” nuisance regressor as in GLM1 showing the activity in pMFC and premotor cortex (p < 0.05, cluster = 1281> threshold = 911) and in (b) with considering “vstim-off” nuisance regressor, GLM3 (no significant activity with p < 0.05). Download Figure 4-1, TIF file.

Since in this task the process of evidence accumulation was fully overlapping with stimulus presentation, and in particular the fact that decision termination could be concurrent with stimulus offset, one may consider the event of stimulus offset as a nuisance factor. Indeed, it is shown that stimulus offset can evoke additional responses in the brain (Herdener et al., 2009; Mullinger et al., 2013, 2017). Therefore, we repeated the previous analysis by adding the “vstim-off” regressor (stick functions at offset times; Extended Data Fig. 3-1*a*) as an additional nuisance factor to ensure that the EEG-related activations (especially those related to EEG energy) are not simply explained by temporal dynamics of sensory information on the screen. In this case, the first-step GLM consists of the four nuisance regressors plus EEG (Table 1, GLM3). Then we regressed its residuals over the EEG energy (Table 1, GLM4). Notably in this condition, the activation map for the EEG energy was similar to the case without inclusion of “vstim-off” (GLM2; Fig. 3) but with positive correlation passing the cluster correction threshold only in the right GC (Fig. 4; *p* < 0.05, cluster-corrected, Table 2). This suggests that at least part of the positive activity seen in relation to EEG energy was not explainable by the “vstim-off” regressor. Given the intrinsic correlation between the two regressors (EEG energy and raw EEG), placing the EEG energy regressor in the second GLM, allows for the primary regressors including the raw EEG signal to absorb the biggest possible variance in the BOLD data leaving only residuals orthogonal to the primary regressors to be explained by the EEG energy. This method is in favor of the null hypothesis (sufficiency of raw EEG regressor) and puts the hardest constraint against adding the EEG energy regressor. Nevertheless, we find essentially the same result if we were to simultaneously use EEG and EEG energy in the second GLM (Extended Data Fig. 4-2; *p* < 0.05, cluster-corrected, Table 2). Once again, the areas within the GC show significant positive activation with EEG energy.

10.1523/ENEURO.0006-22.2022.f4-2 Extended Data Figure 4-2 Robustness of the activity in the right GC for simultaneous regression with raw EEG and EEG energy regressors in the second-step GLM (GLMs 5, 6). Group-average activation map (t stats) for the “EEG energy” regressor in GLM6 showing activity in the insular, opercular, and inferior somatosensory cortices (p < 0.05, cluster-corrected (cluster = 213 > threshold = 146). a, Axial and multiple coronal views. b, Lateral and medial views on the inflated cortex. Download Figure 4-2, TIF file.

10.1523/ENEURO.0006-22.2022.f4-3 Extended Data Figure 4-3 Activations revealed by the raw EEG and EEG energy when using a boxcar function for the duration of visual stimulus (GLMs 7–9). Group-average activation map (t stats) for (a) the “EEG energy” regressor in GLM9 (p < 0.05, cluster-corrected, cluster = 219 > threshold = 138) and (b) the raw EEG regressor in GLM8 with no significant (p < 0.05) activity. Download Figure 4-3, TIF file.

On the other hand, inclusion of “vstim-off,” removed the previously reported activation in pMFC in relation to the “raw EEG” altogether (Extended Data Fig. 4-1*b*), suggesting that the observed activity in pMFC could be because of visual offset rather than choice process per se. Indeed, examination of activation maps for all four nuisance factors show a significant cluster for visual offset that overlaps with pMFC (Extended Data Fig. 3-2*b*). A qualitatively similar result was obtained if one were to use a boxcar for the duration of stimulus presentation as the nuisance factor instead of “vstim-off” (GLM7). In this case again a significantly positive correlate in the right GC was observed for EEG energy (GLM9, Extended Data Fig. 4-3*a*; *p* < 0.05, cluster-corrected, Table 2). Once again, no significant cluster of activity related to the raw EEG was observed in this case when stimulus duration is taken into account (GLM8; Extended Data Fig. 4-3*b*). These results suggest that the observed positive activations in the GC is not explainable by stimulus dynamics and are most likely reflecting the process of value-based decision-making.

**Figure 5.**
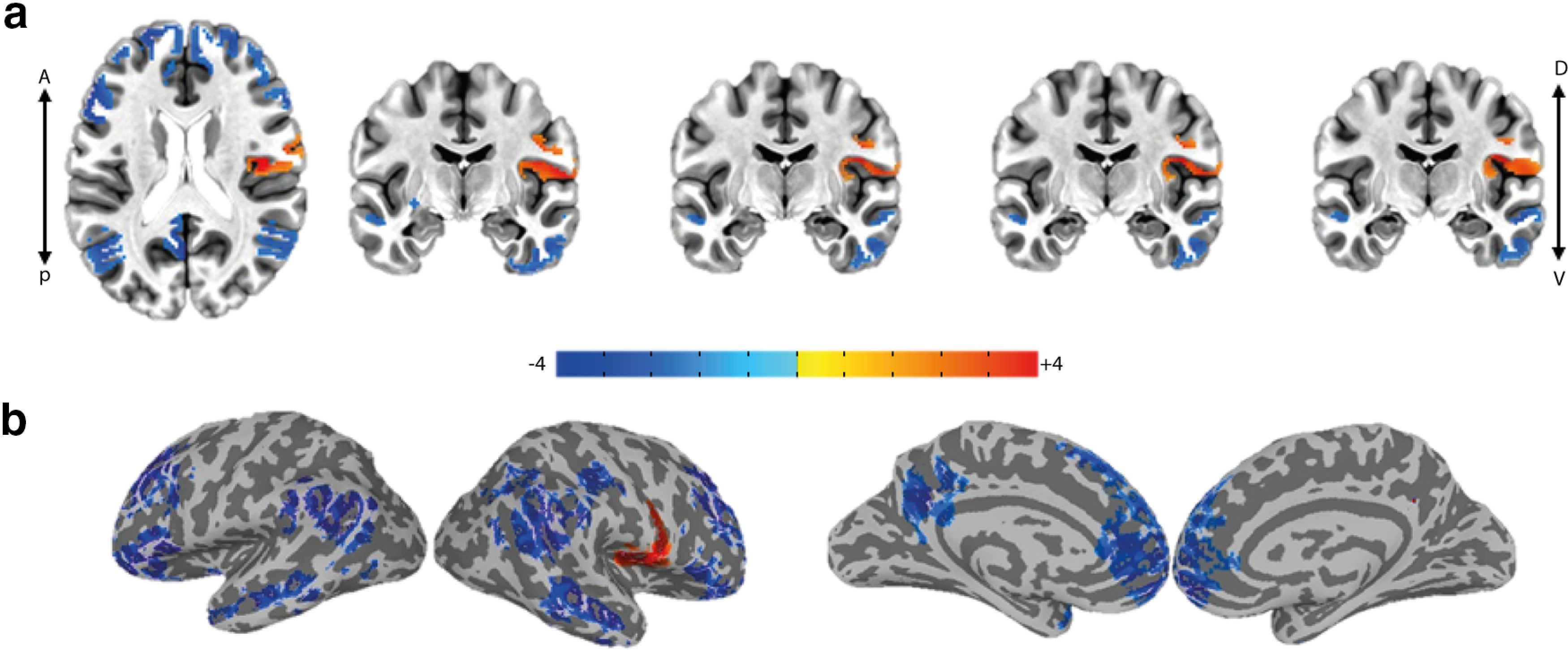
Right GC activation is robust to normalization of EEG-driven regressors (GLMs 10–11). Group-average activation map (*t* stats) for the normalized “EEG energy” regressor in GLM11 showing activity in the insular, opercular and inferior somatosensory cortices (*p* < 0.05, cluster-corrected (cluster = 567 > threshold = 295); ***a***, axial and multiple coronal views, ***b***, lateral and medial views on the inflated cortex. See Extended Data Figure 5-1 for variability of average energy of EEG over a total run between subjects.

Moreover, the EEG power for different subjects may vary substantially because of multiple reasons such as intrinsic differences in neuronal activity levels, differences in the lead-field gains or measurement noise. Therefore, it is often recommended to normalize the EEG signal of subjects to achieve a more reliable group-level inference (Cohen, 2014). Here, also, we see a relatively wide dynamic range in mean EEG power across subjects (Extended Data Fig. 5-1). In order to make sure that such variability does not affect our main conclusions about significant positive activations observed in the right GC, we performed another analysis with normalized EEG regressors (Table 1, GLMs 10–11; for details, see Materials and Methods) on the residuals of GLM5. Results show that EEG normalization reproduced the significant positive activation in the right GC in relation to EEG energy even to a wider extent (GLM11, Fig. 5; *p* < 0.05, cluster-corrected, Table 2). Once again, we did not find any significant activity for the normalized raw EEG regressor in this case (GLM10). Nevertheless, some of the variability in variance of EEG regressors across subjects may indeed reflect true differences in the task-related activity across their brains, in which case using normalized regressors is expected to reduce the group level significance.

10.1523/ENEURO.0006-22.2022.f5-1 Extended Data Figure 5-1 Between subject variability of average energy of the “raw EEG” over a whole run. a, Average energy of the electric potential over all decision periods in an experimental run corresponding to the best electrode of each subject. b, Histogram of average energy of subjects’ best electrode EEG Download Figure 5-1, TIF file.

### Absence of significant cluster correlates with higher powers of EEG signal

**Figure 6.**
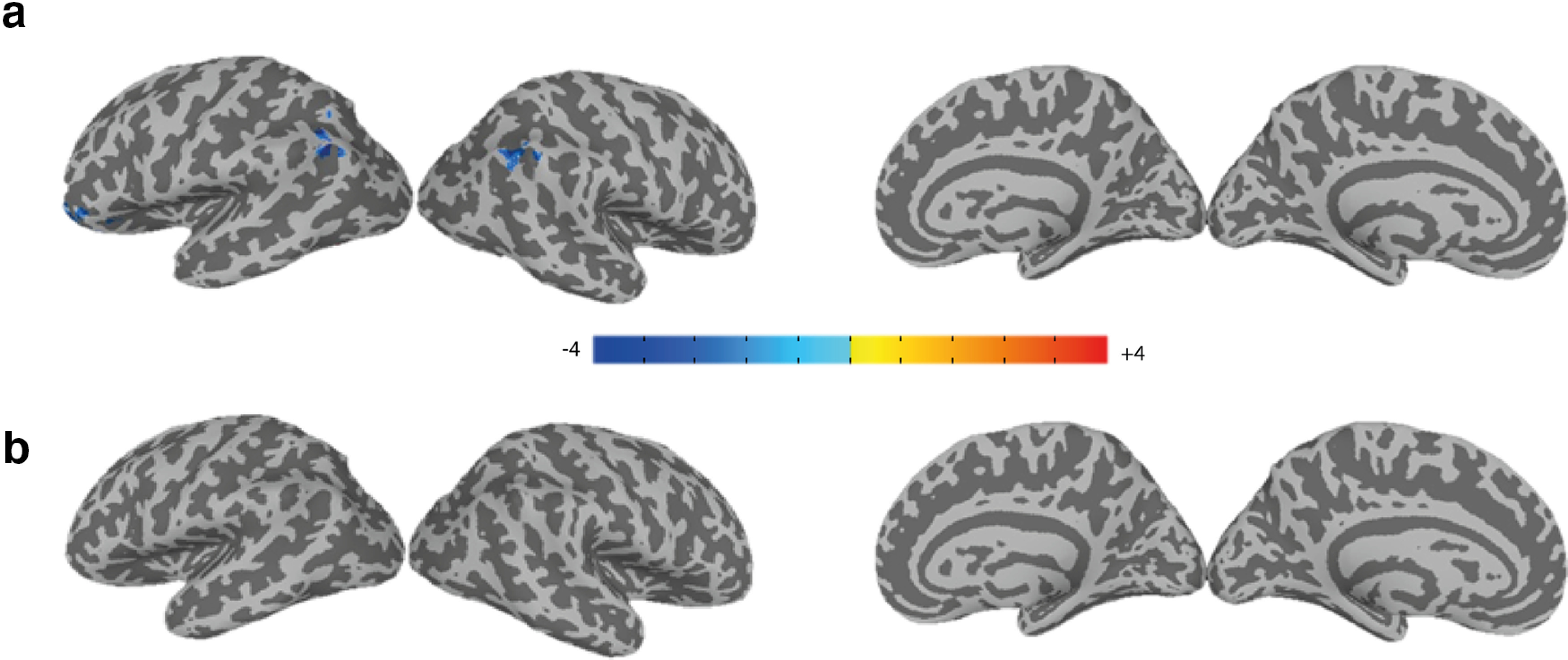
Higher order EEG powers 3 and 4 do not reveal significant positive clusters of brain activation during food choice (GLMs 12–13). Group-average activation map (*t* stats) for higher powers of EEG in GLM12 and GLM13. ***a***, Negative correlations with EEG pow3 (*p* < 0.05, cluster-corrected; clusters > threshold = 121). ***b***, No significant correlations with EEG pow4 (*p* < 0.05, cluster-corrected; cluster threshold = 71).

Given the highly nonlinear mapping between electrical activity in a voxel and its BOLD signal such as those predicted by the Balloon model (Buxton et al., 1998), it is plausible that still higher order nonlinearities have to be considered in EEG-informed fMRI analysis. In order to check for possible higher order nonlinearities, we created regressors for higher powers of the EEG (powers 3 and 4). To account for the multicollinearity among various powers of EEG, analysis of the power 3 of EEG (GLM12) was performed on the residual of first-step GLM which included nuisance factors and the EEG regressor (GLM3). For power 4 of EEG, we did the regression (GLM13) on the residuals of GLM4 because of high correlation between power 2 (EEG energy) and power 4. Notably, no significant positive cluster of correlations with higher powers of EEG (powers 3 and 4) was found (Fig. 6; *p* > 0.05, cluster-corrected). Some clusters of negative activation related to power 3 of EEG was found close to prefrontal and temporoparietal areas (Fig. 6; Table 2).

Finally, we did not observe any subcortical activations with any of the EEG-driven regressors used (raw EEG or any of its powers). The only notable subcortical activation was found in the amygdala in correlation with the “value difference” regressor (Extended Data Fig. 3-2*c*).

## Discussion

EEG-informed fMRI analysis is a promising method in localizing fast cognitive processes in the brain such as the formation of a decision. Here, using simultaneous EEG-fMRI in a value-based decision-making task, revealed significant correlates of evidence accumulation in the insular and opercular cortices. This activity was uncovered by using “EEG energy” as the EEG-driven regressor and was missed if one were to use the raw EEG correlate of DV as was done previously (Pisauro et al., 2017). Here, we proved the relevance of EEG energy for BOLD theoretically, in agreement with the previous experimental evidence (Wan et al., 2006). Notably, despite the highly nonlinear nature of neurovascular coupling, we did not find any significant correlations with the higher powers of EEG (powers 3 and 4) in this task.

Given the multicollinearity of the experimental procedure at hand in which the decision formation and stimulus presentations were concurrent, one needed to make sure that the EEG-related brain correlates were not because of stimulus onset, duration and offset. This problem was addressed by using step-wise GLMs which lets the nuisance regressors to describe as much as the variance in the BOLD as they can and leave the orthogonal components to be described by the regressor of interest in the second-step GLM (GLMs 2, 4–6, 8–13). Using this orthogonalization procedure, the “EEG energy” as the BOLD regressor revealed activity in the right operculum, insula and the inferior somatosensory cortex (Figs. 3-5). Multiple parts of insula and operculum, including the anterior, middle and posterior insula as well as the frontal and parietal operculum (PO) extending to the inferior somatosensory cortex (area 3b) are reported to participate in taste and gustatory representations (Small, 2010; Veldhuizen et al., 2011; Mai and Paxinos, 2012) and form the GC. Some meta-analysis studies (Small, 2010; Yeung et al., 2018) have distinguished the involvement of these diverse cites, in various aspects of taste and gustatory processing. The middle insula is reported for its role in coding the “pleasantness” aspect of taste and in attention to taste (Small, 2010) as well as its participance in coding the affective value and quality of food regardless of its intensity (Yeung et al., 2018). The activity observed in the middle insula in our study, agrees well with the hypothesized role of insula in this task which is to evaluate the food’s pleasantness. It is conceivable for the activity in the GC to induce a gustatory imagery of the food items which are used in the evidence accumulation process for food choice. Interestingly, the insular and opercular regions have been also previously reported to be implicated in “gustatory imagery” (Kobayashi et al., 2004, 2011). Furthermore, the structural (Ogawa, 1994; Ghaziri et al., 2018) and functional (Roy et al., 2009) connectivity between insula and amygdala and the fact that amygdala showed value-based activation in this task is in agreement with a possible value retrieval from amygdala. Representation of the subjective value of choice items in the relevant primary sensory cortex is also previously reported (Shuster and Levy, 2018).

Experimental evidence for a quadratic relation between the vascular input and the neural electrical sources estimated from EEG was provided previously (Wan et al., 2006) and used for studies involving epileptic patients (Murta et al., 2015; Abreu et al., 2018). Here, we extended the use of EEG energy to cognitive studies and argue from a theoretical standpoint that “EEG energy” should be a better correlate of BOLD response compared with EEG signal itself for the use in EEG-informed fMRI analyses. Consistent with this suggestion, correlations between the BOLD response and the power of EEG in the alpha band were reported in some studies especially in the resting state experiments (de Munck et al., 2009; Sato et al., 2010). Correlations between the BOLD response and various frequency bands of EEG in task-based experiments are also investigated (Scheeringa et al., 2009; Sato et al., 2010). A negative correlation between the theta power and BOLD response in the areas of the default mode network (DMN) is reported by Scheeringa et al. (2009). Actually, the negatively correlated regions with the EEG energy regressor in our study also highly overlap with the DMN (Table 2), and this is plausible since the lower frequency bands of EEG (including theta band) dominate in EEG spectrum. These observed negative correlations with EEG energy during the decision-making may suggest shutting down of these areas during value-based decision-making.

The nonlinear nature of the neurovascular coupling could engender BOLD correlation with still higher powers of EEG signal. However, examining powers 3 and 4 of EEG signal in this study did not reveal any activations across the brain, suggesting “EEG energy” as a suitable and sufficient correlate of the BOLD response at least for this data.

Furthermore, we shall note that despite the observed significant activity in amygdala in correlation with the “value difference” regressor, there was no significant subcortical clusters in positive correlation with the EEG-driven regressors. This may indicate that the subcortical regions do not play a role in the process of evidence accumulation but only provide the needed inputs (value memory) for cortical regions responsible for decision-making. On the other hand, this negative result may also be due the substantially lower signal-to-noise-ratio of the subcortical potentials on the EEG signal recorded on the scalp.

In summary, we conclude that because of the nonlinear relation between EEG and fMRI, “EEG energy” (or total power) proves critical for EEG-informed fMRI analysis. In particular, using EEG energy regressor in GLM of value-based decision-making revealed evidence accumulation activity in the operculum, insula and inferior somatosensory cortex. Activity in these regions as parts of the GC indicates that gustatory imagery is likely to be used during the decision-making process for food choices and implicates cortical areas traditionally involved in palatability processing, in value-based decision-making. Further investigations using electrophysiological techniques in human or non-human primates can help elucidate the exact dynamics of evidence accumulation in the gustatory areas during food choice.

## Synthesis

Reviewing Editor: Alexander Soutschek, Ludwig-Maximilians-Universitat Munchen Decisions are customarily a result of the Reviewing Editor and the peer reviewers coming together and discussing their recommendations until a consensus is reached. When revisions are invited, a fact-based synthesis statement explaining their decision and outlining what is needed to prepare a revision will be listed below. The following reviewer(s) agreed to reveal their identity: Sally Eldeghaidy.

The authors present an improved methodology to a previously published work by Pisauro et al. (2017) to uncover activity within the gustatory cortex during food choice process. The reviewers agree that the manuscript is well written throughout and addresses an interesting topic. However, the reviewers raised a couple of concerns which the authors need to address before the manuscript can be accepted for publication.

1. The reviewers suggest adding a brief summary of the food choice paradigm used in Pisauro et al 2017. Although this is illustrated in Figure 1, a summary of the task will be beneficial.

2. In addition, in line 88 page 3 the authors indicate that they used cleaned EPI images for MNI registration process, please explain how they have been cleaned (e.g. motion correction).

3. It is unclear why the authors regressed the residuals of the first GLM on the EEG energy variable instead of entering both the raw EEG data and the energy predictor simultaneously into a GLM. Are the findings robust to using the suggested alternative approach?

4. A total of twelve GLMs were computed. The authors should describe these analyses in more detail in the Materials and Methods section.

5. Please revise the formatting of Figure 2 - parts of the figure seem to be cut off in the manuscript.

6. On page 6, the “vstim-off” regressor comes out of the blue. Please introduce this regressor in more detail so that readers can understand the rationale behind these analyses.

7. The authors should also provide more details when reporting fmri results in main text, for example the peak coordinates or number of significant voxel in a cluster.

8. The authors write that the codes for simulating the cluster thresholds are accessible via a direct email to the corresponding author. Please add a separate “code accessibility” section at the end of the methods section with a link to the code on a public repository (e.g., OSF).

9. In table 2, please add also the labels (e.g., gustatory cortex) and the Brodmann areas to the reported clusters.

## Author Response

Dear Colleagues,

We would like to thank the editor for organizing the review process and the reviewers for their comments which have resulted in an improved manuscript. As a result of comments, we have done an additional GLM analysis and added its result as a new supplementary figure 4-2 and have updated the tables and figure legends. We have also highlighted all major changes in the text. Tables are also updated and completed based on the comments. Below are our point-by-point responses to the comments.

Regards,

Ali Ataei, Arash Amini and Ali Ghazizadeh

Synthesis of Reviews:

The authors present an improved methodology to a previously published work by Pisauro et al. (2017) to uncover activity within the gustatory cortex during food choice process. The reviewers agree that the manuscript is well written throughout and addresses an interesting topic. However, the reviewers raised a couple of concerns which the authors need to address before the manuscript can be accepted for publication.

1. The reviewers suggest adding a brief summary of the food choice paradigm used in Pisauro et al 2017. Although this is illustrated in Figure 1, a summary of the task will be beneficial.

Additional explanation of the task is added to Result section page 5 lines 17-23:

"Each trial began with display of a fixation point for a random time in the range 2-4 s and the subjects were asked to maintain the fixation point. Then, two randomly selected food items were displayed to the right and to the left of the fixation point for 1.25 s and the subject had to respond during this period. The difficulty of the decision was controlled by the value difference (VD) in the ratings of the presented items. Before the experiment and outside the scanner, each subject was asked to rate 80 snack items with real scores in the range of (-5,5) based on his/her subjective value. “

2. In addition, in line 88 page 3 the authors indicate that they used cleaned EPI images for MNI registration process, please explain how they have been cleaned (e.g. motion correction).

We have added the preprocessing steps already done on the EPI images in page 3 lines 4-6:

” ... which included motion-correction, slice-time correction, high-pass filtering (> 100 s) and spatial smoothing (8mm FWHM).”

3. It is unclear why the authors regressed the residuals of the first GLM on the EEG energy variable instead of entering both the raw EEG data and the energy predictor simultaneously into a GLM. Are the findings robust to using the suggested alternative approach?

Thank you for the comment. Given the intrinsic correlation between the two regressors (EEG energy and raw EEG), placing the EEG energy regressor in the second GLM, allows for the primary regressors including the raw EEG signal to absorb the biggest possible variance in the BOLD data leaving only residuals orthogonal to the primary regressors to be explained by the EEG energy. This method is in favor of the null hypothesis (sufficiency of raw EEG regressor) and puts the hardest constraint against adding the EEG energy regressor. This step was necessary since we wanted to ensure that addition of EEG energy really explains changes in brain activation over and beyond raw EEG. Nevertheless, our results are robust to the scenario where we put both regressors in a same GLM. In all cases the gustatory cortex is significantly active for the EEG energy regressor and passes the cluster-correction threshold.

To address the reviewers comment we have added the results of this regression to supplemental Fig 4-2 and added a description to result section page 8 line 16-25:

” Given the intrinsic correlation between the two regressors (EEG energy and raw EEG), placing the EEG energy regressor in the second GLM, allows for the primary regressors including the raw EEG signal to absorb the biggest possible variance in the BOLD data leaving only residuals orthogonal to the primary regressors to be explained by the EEG energy. This method is in favor of the null hypothesis (sufficiency of raw EEG regressor) and puts the hardest constraint against adding the EEG energy regressor. Nevertheless, we find essentially the same result if we were to simultaneously use EEG and EEG energy in the second GLM. (Fig 4-2; p-value < 0.05, cluster-corrected, Table 2). Once again, the areas within the Gustatory cortex show significant positive activation with EEG energy.”

We have added this new GLM to Table 1 (please note the new arrangement and numbering of GLMs in this table).

The caption for the new figure 4_2 is added to the “Extended data Legends” section in page 15, lines 30-35.

4. A total of twelve GLMs were computed. The authors should describe these analyses in more detail in the Materials and Methods section.

All GLMs were explicitly described in the “Results” section while the associated mathematical and computational details are described in the materials and methods. However, to better clarify the logic and procedures taken we have added more details in the Materials and Method section under the heading “fMRI analysis & the generalized linear models “ in page 3, lines 30-37 and page 4, lines 1-17 :

"We used step-wise GLM analyses in order to gauge robustness of our findings and to overcome the multicollinearity between various EEG-driven regressors (various powers of EEG) as well as the correlation between the regressor of interest and the nuisance regressors. For most stringent analyses, we used EEG raw signal in the primary GLM and the higher powers in the subsequent GLMs to ensure explaining brain activation over and beyond raw EEG. GLM1 is just same as the main GLM analysis in (Pisauro et al. 2017) and consists of the three nuisance regressors and the raw EEG regressor. GLM 2 is a regression of the residuals of GLM1 over the “EEG energy”. This paradigm is in favor of the null hypothesis (sufficiency of raw EEG) and lets it explain as much variance in the fMRI data and only leaves the orthogonal components to be explained by the new regressor, i.e. EEG energy. Since visual stimulus offset timing is close to the reaction times for food choice decision (< 1s), it can be a serious confound for the decision correlates observed in brain activation. To control for this effect, GLM3 is performed. This GLM is similar to GLM1, but with an added nuisance regressor accounting for the stimulus offset. Once again GLM4 regresses the residuals of GLM3 over the EEG energy to ensure robustness of findings with respect to inclusion of vstim-off. Simultaneous regression using both raw EEG and EEG energy is also performed (GLM6) over residuals of GLM5 which only consists of the nuisance regressors. We have also performed similar analyses replacing vstim-off with a boxcar function for the duration of visual stimulus in GLMs 7-9. Since the total EEG power substantially differs from subject to subject, we repeated similar analyses in GLMs 10-11, using normalized EEG-driven regressors (normalizing EEG regressors in each subject by their standard deviation). Higher order powers of EEG were also investigated. GLM12 regresses the residuals of GLM3 over the third power of EEG signal. In order to test the fourth power of EEG signal (GLM13), we used residuals of GLM4 rather than GLM3, due to the high correlation between powers 2 and 4.”

5. Please revise the formatting of Figure 2 - parts of the figure seem to be cut off in the manuscript.

Thank you for pointing this out we have corrected the embedded figure 2.

6. On page 6, the “vstim-off” regressor comes out of the blue. Please introduce this regressor in more detail so that readers can understand the rationale behind these analyses.

We have added some more explanation and the logic for inclusion of vstim-off in page 8, lines 5-8:

"one may consider the event of stimulus offset as a nuisance factor. Indeed, it is shown that stimulus offset can evoke additional responses in the brain (Herdener et al. 2009; Mullinger et al. 2013, 2017). Therefore, we repeated the previous analysis by adding the ‘vstim-off’ regressor (stick functions at offset times, Figure 3-1a) as an additional nuisance factor to ensure that the EEG related activations (especially those related to EEG energy) are not simply explained by temporal dynamics of sensory information on the screen.”

7. The authors should also provide more details when reporting fmri results in main text, for example the peak coordinates or number of significant voxel in a cluster.

The numbers of significant voxels were already mentioned in the captions of the corresponding figures for each reported result. Table 2 is now updated and contains the peak coordinates for all reported results.

8. The authors write that the codes for simulating the cluster thresholds are accessible via a direct email to the corresponding author. Please add a separate “code accessibility” section at the end of the methods section with a link to the code on a public repository (e.g., OSF).

The code accessibility section is added in page 5 lines 10-12:

The lead-filed matrix and the simulation code as well as the fMRI analysis codes are available at https://github.com/poyaata/code-data .

9. In table 2, please add also the labels (e.g., gustatory cortex) and the Brodmann areas to the reported clusters.

This information is added to Table 2.
