# Supplementary figures and images for: Gustatory Cortex Is Involved in Evidence Accumulation during Food Choice
Source: eNeuro. 2022 May 17;9(3):ENEURO.0006-22.2022. doi: 10.1523/ENEURO.0006-22.2022 (PMC9121914; doi:10.1523/ENEURO.0006-22.2022)

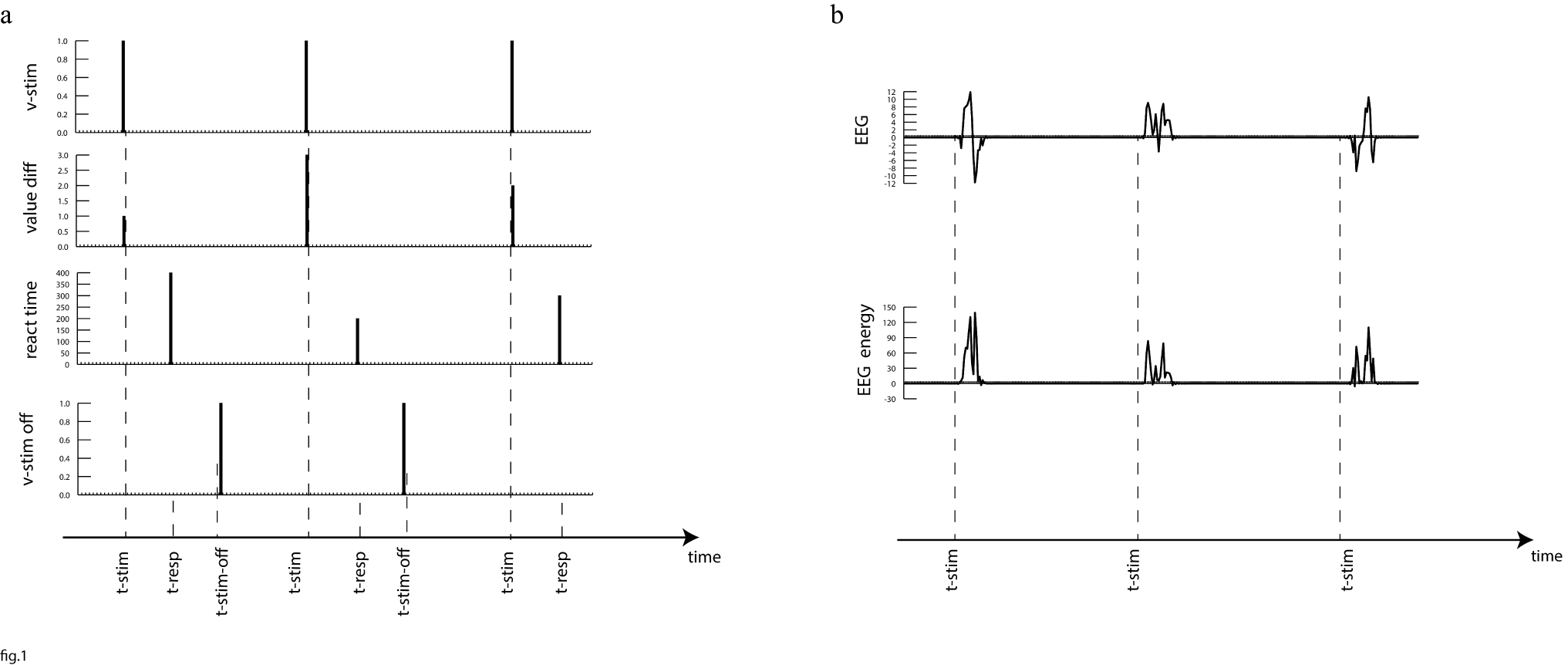

Supplement: Extended Data Figure 3-1 — The regressors used in fMRI analyses. a, The four nuisance regressors; the visual onset, the value difference, the reaction time, and the visual offset regressors. b, The regressors of interest; the raw EEG and the EEG energy regressors. Download Figure 3-1, TIF file. [file enu-eN-NWR-0006-22-s02.tif]

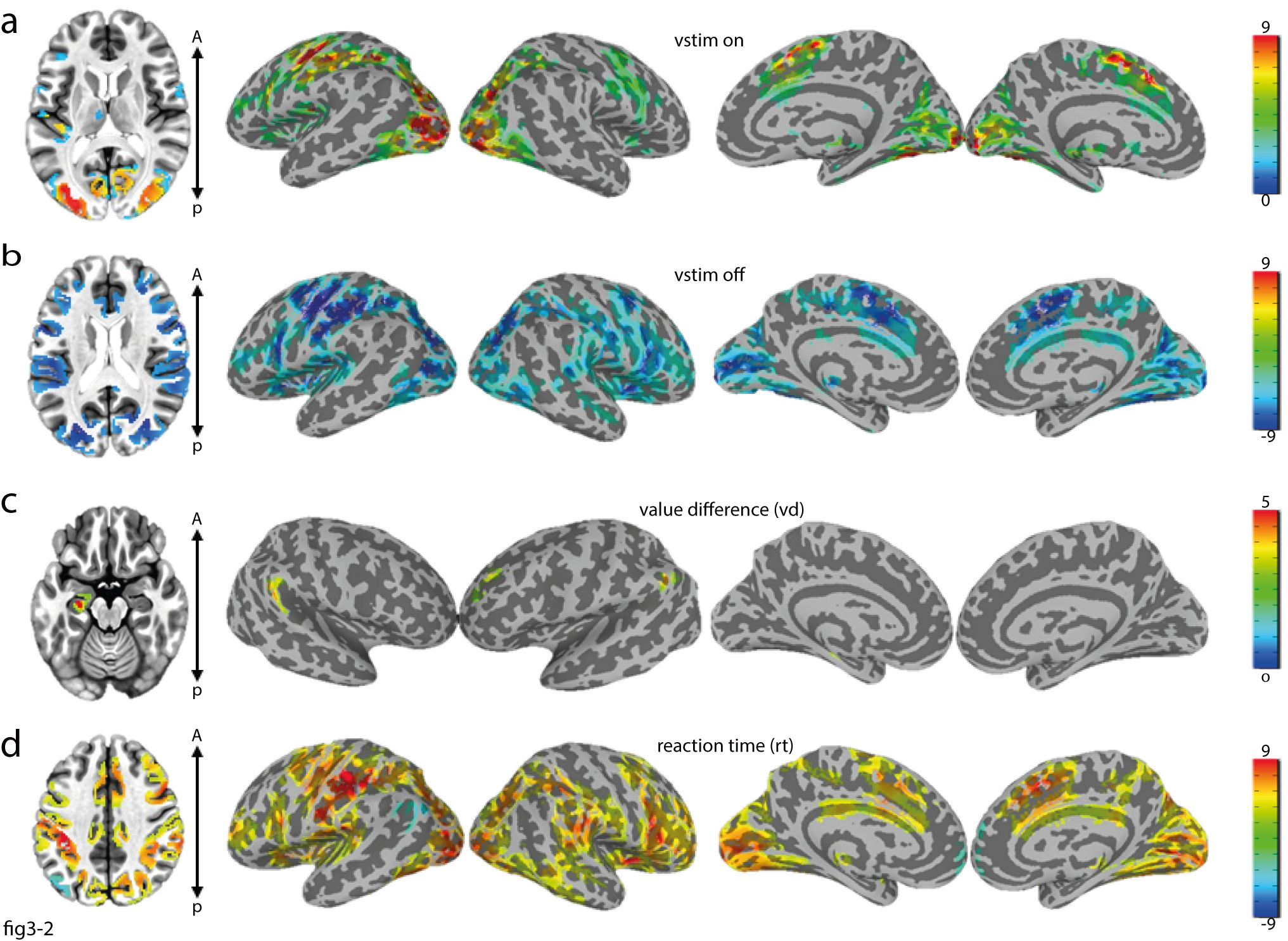

Supplement: Extended Data Figure 3-2 — Activation maps for the nuisance regressors (GLM 3). Group-average activation map (t stats) for the nuisance regressors all with p < 0.01 and cluster-corrected: (a) visual onset (vstim) regressor, (b) visual offset (vstim-off) regressor, (c) value difference (Vd) regressor, and (d) reaction time (rt) regressor. Download Figure 3-2, TIF file. [file enu-eN-NWR-0006-22-s03.tif]

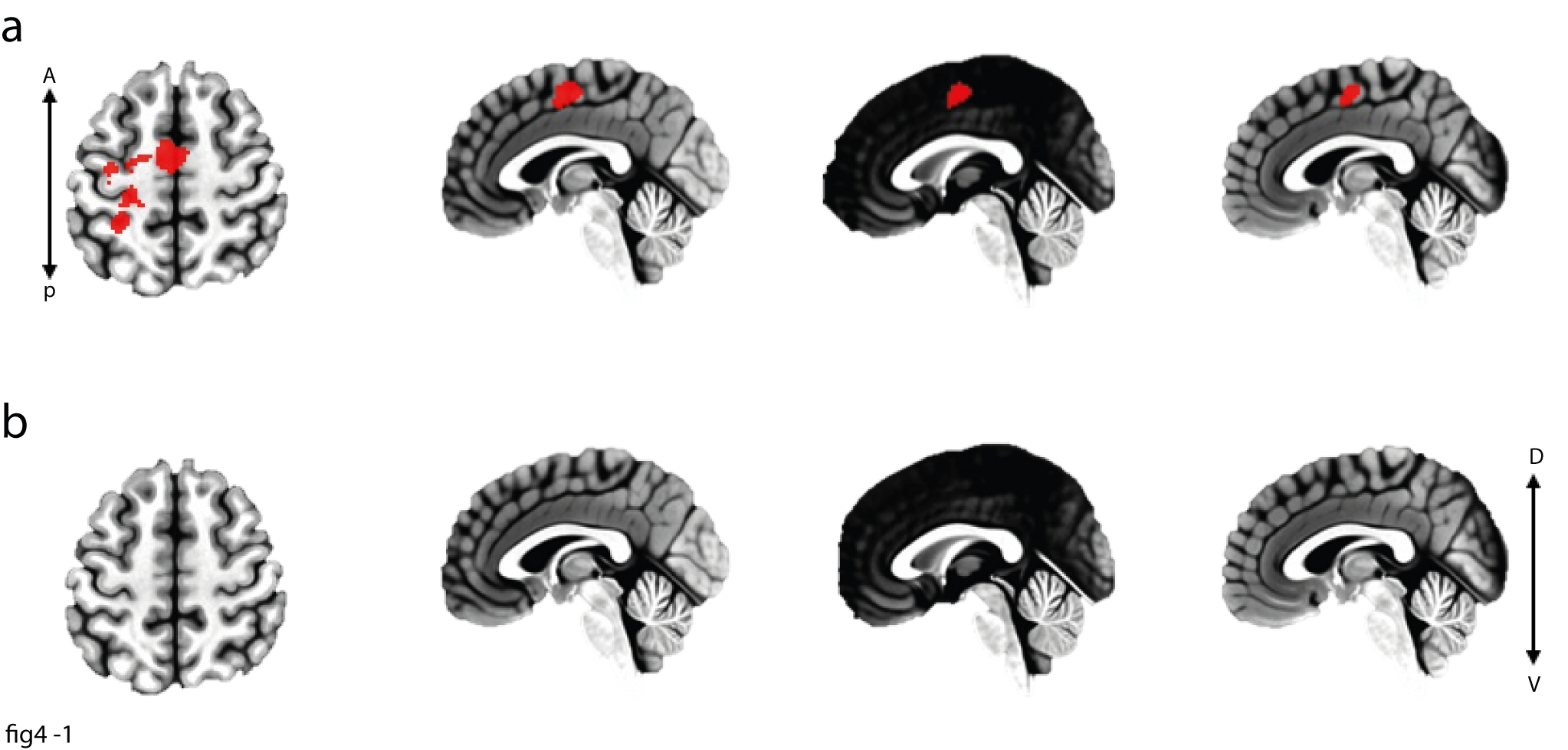

Supplement: Extended Data Figure 4-1 — Comparing the activity map for the raw EEG regressor with and without considering stimulus offset as a nuisance regressor (GLMs 1, 3) Group-average activation map for the raw EEG regressor (a) without considering “vstim-off” nuisance regressor as in GLM1 showing the activity in pMFC and premotor cortex (p < 0.05, cluster = 1281> threshold = 911) and in (b) with considering “vstim-off” nuisance regressor, GLM3 (no significant activity with p < 0.05). Download Figure 4-1, TIF file. [file enu-eN-NWR-0006-22-s04.tif]

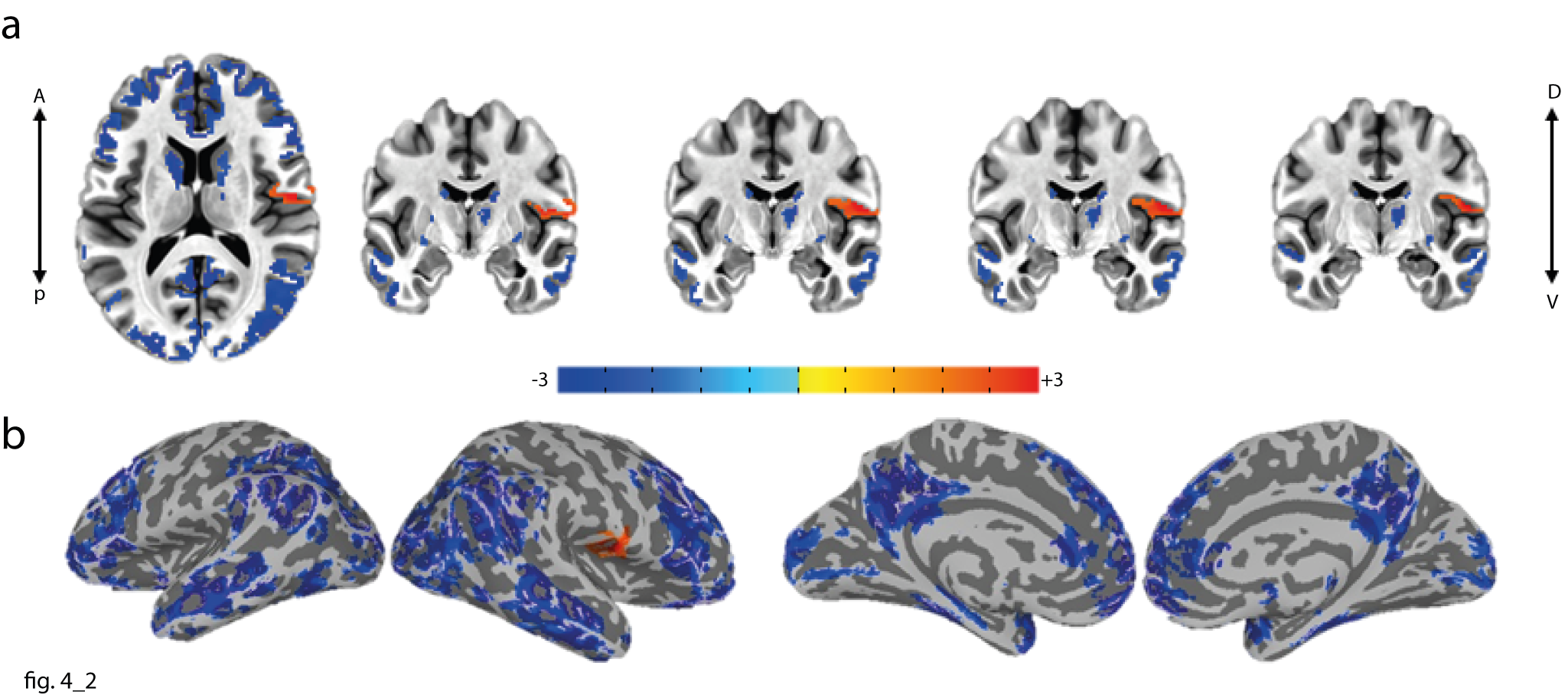

Supplement: Extended Data Figure 4-2 — Robustness of the activity in the right GC for simultaneous regression with raw EEG and EEG energy regressors in the second-step GLM (GLMs 5, 6). Group-average activation map (t stats) for the “EEG energy” regressor in GLM6 showing activity in the insular, opercular, and inferior somatosensory cortices (p < 0.05, cluster-corrected (cluster = 213 > threshold = 146). a, Axial and multiple coronal views. b, Lateral and medial views on the inflated cortex. Download Figure 4-2, TIF file. [file enu-eN-NWR-0006-22-s05.tif]

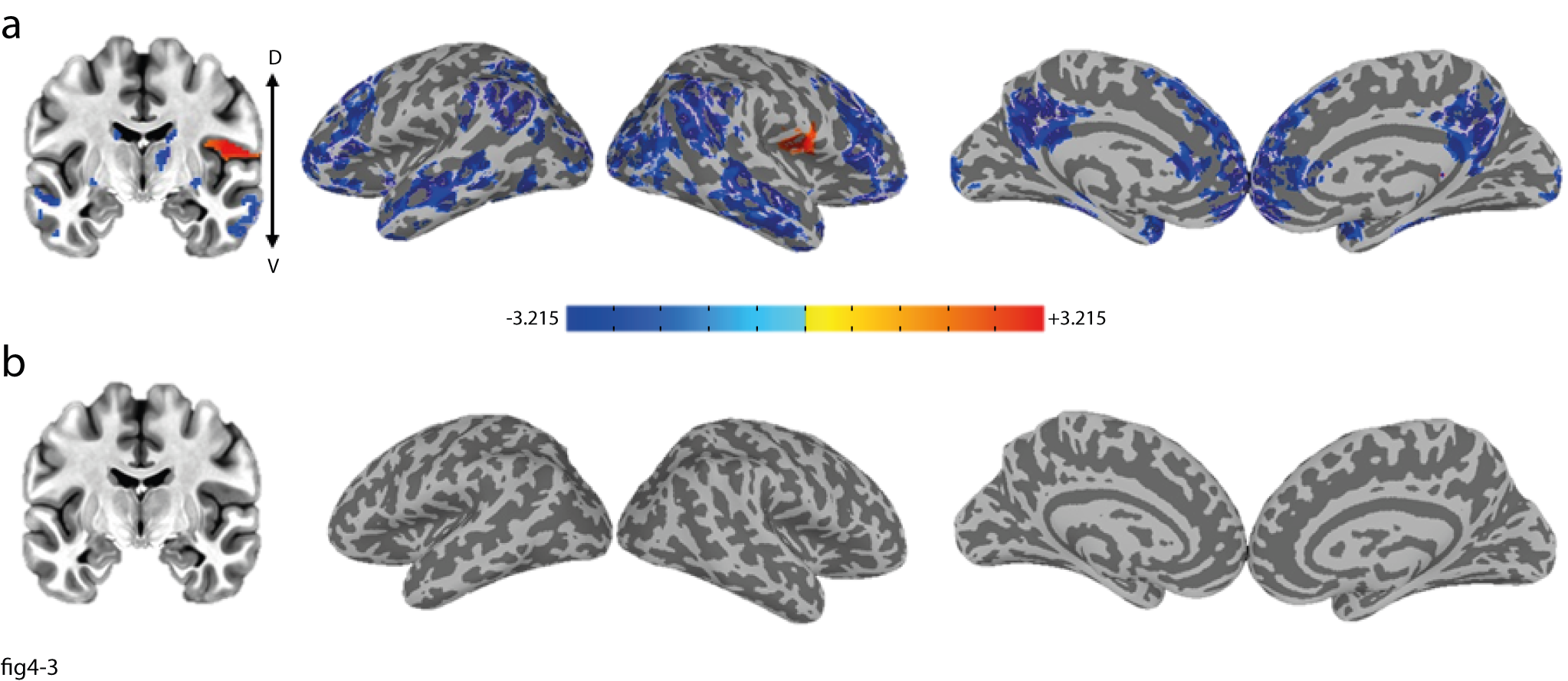

Supplement: Extended Data Figure 4-3 — Activations revealed by the raw EEG and EEG energy when using a boxcar function for the duration of visual stimulus (GLMs 7–9). Group-average activation map (t stats) for (a) the “EEG energy” regressor in GLM9 (p < 0.05, cluster-corrected, cluster = 219 > threshold = 138) and (b) the raw EEG regressor in GLM8 with no significant (p < 0.05) activity. Download Figure 4-3, TIF file. [file enu-eN-NWR-0006-22-s06.tif]

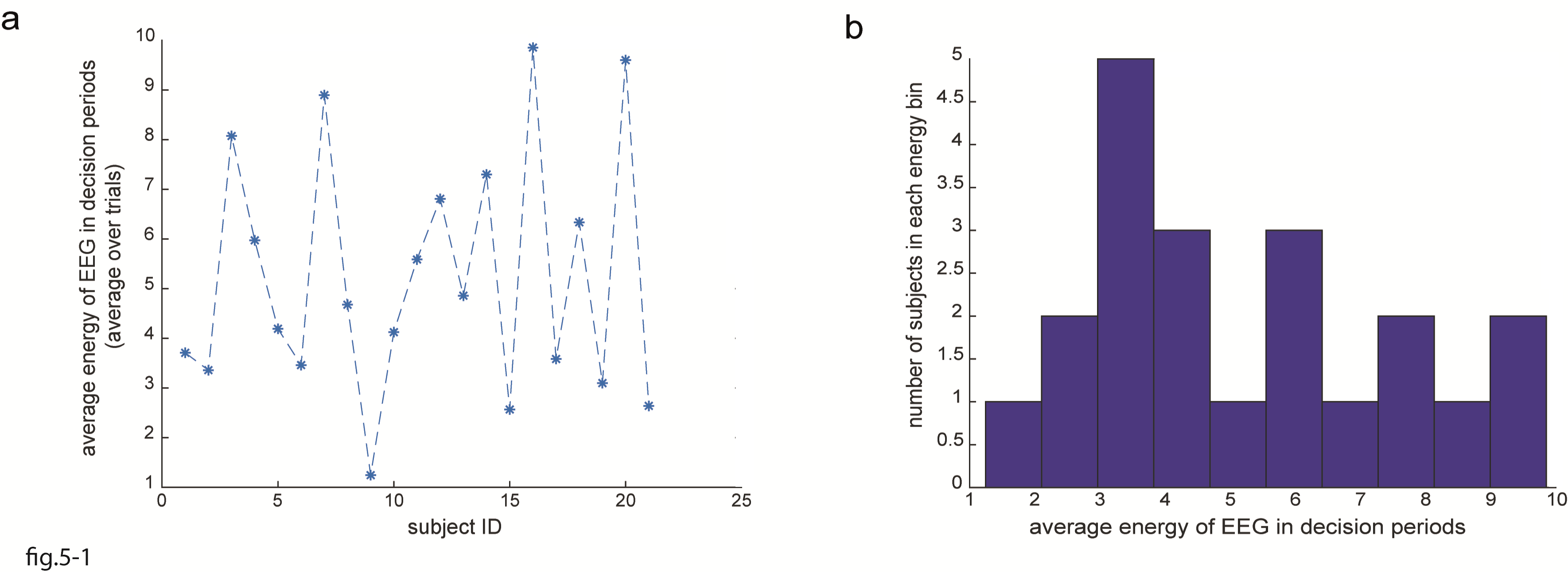

Supplement: Extended Data Figure 5-1 — Between subject variability of average energy of the “raw EEG” over a whole run. a, Average energy of the electric potential over all decision periods in an experimental run corresponding to the best electrode of each subject. b, Histogram of average energy of subjects’ best electrode EEG Download Figure 5-1, TIF file. [file enu-eN-NWR-0006-22-s07.tif]
